# Ultrathin Titanium Dioxide Coating Enables High-Rate and Long-Life Lithium Cobalt Oxide

**DOI:** 10.3390/ma17123036

**Published:** 2024-06-20

**Authors:** Liu Gao, Xin Jin, Zijin Li, Fujie Li, Binghui Xu, Chao Wang

**Affiliations:** Institute of Materials for Energy and Environment, College of Materials Science and Engineering, Qingdao University, Qingdao 266071, China

**Keywords:** lithium cobalt oxide, atomic layer deposition, surface coating, titanium dioxide, lithium-ion batteries

## Abstract

Lithium cobalt oxide (LCO) has been widely used as a leading cathode material for lithium-ion batteries in consumer electronics. However, unstable cathode electrolyte interphase (CEI) and undesired phase transitions during fast Li^+^ diffusivity always incur an inferior stability of the high-voltage LCO (HV-LCO). Here, an ultra-thin amorphous titanium dioxide (TiO_2_) coating layer engineered on LCO by an atomic layer deposition (ALD) strategy is demonstrated to improve the high-rate and long-cycling properties of the HV-LCO cathode. Benefitting from the uniform TiO_2_ protective layer, the Li^+^ storage properties of the modified LCO obtained after 50 ALD cycles (LCO-ALD50) are significantly improved. The results show that the average Li^+^ diffusion coefficient is nearly tripled with a high-rate capability of 125 mAh g^−1^ at 5C. An improved cycling stability with a high-capacity retention (86.7%) after 300 cycles at 1C is also achieved, far outperforming the bare LCO (37.9%). The in situ XRD and ex situ XPS results demonstrate that the dense and stable CEI induced by the surface TiO_2_ coating layer buffers heterogenous lithium flux insertion during cycling and prevents electrolyte, which contributes to the excellent cycling stability of LCO-ALD50. This work reveals the mechanism of surface protection by transition metal oxides coating and facilitates the development of long-life HV-LCO electrodes.

## 1. Introduction

LiCoO_2_ (LCO) is the most important cathode material for lithium-ion batteries (LIBs) in consumer electronics due to its high volumetric energy density [1,2]. As a typical α-NaFeO_2_ layered material, LCO enables a high theoretical capacity of 274 mAh g^−1^ [3]. Unfortunately, only 165 mAh g^−1^ can be achieved for present LCO with a charging voltage at 4.35 V, which means that only about 60% of lithium ions (Li^+^) in LCO can be released in practice. By elevating the charging cutoff voltage, the LCO would release more Li^+^ with a high capacity, demonstrating great potential to achieve a high density for LCO-based LIBs [1]. However, when charged above 4.5 V, LCO suffers from a poor cycling stability because of phase transition and an unstable LCO surface [4].

Strategies have been demonstrated to address the above problem of high-voltage LCO (HV-LCO), including nano-structuring [5], surface coating [6,7], electrolyte modification [8] and cation doping [9,10]. Surface coating with highly ionic/electronic conductors or metal oxides can effectively improve the rate performance and long-cycling stability of HV-LCO [10,11,12]. In general, a coated layer can regulate electrode–electrolyte interface compatibility, suppressing the uneven growth of CEI and facilitating charge transfer during charge–discharge cycles [4]. For instance, coating of amorphous alumina on LCO can improve its electronic conductivity and cycling stability [13]. The in situ formed Li_3_AlF_6_ crystal layer suppresses the surface stripping and the as-caused disordered rock-salt phase transition, thus facilitating the stability of LCO during cycling. A surface-modified LCO exhibits a high-rate capability owing to the coating of the high Li^+^ conductive Li_2_Zr(PO_4_)_2_ layer, which can help the formation of stable CEI with a low interface resistance [14]. Overall, the key to surface modification for stable HV-LCO is the construction of high-voltage electrochemically stable coating layers, which should be uniform and beneficial to promote the formation of high-quality CEI.

Among various materials, titanium dioxide (TiO_2_) coating has been widely reported in the surface-modified research owing to its excellent chemical stability and stable corrosion resistance [6,15,16,17,18,19]. For example, Wang et al. prepared an TiO_2_−LiF composite coating on the surface of LCO and significantly improved the chemical stability at a high voltage and enhanced cycling performance [15]. Xu et al. reported that coated TiO_2_ could reduce surface residual lithium and lower the formation of the Co_3_O_4_ and CEI layer due to the transformation to a LiTiO_2_-rich layer after annealing, which was beneficial to an improved cyclic performance [16]. Zhou et al. demonstrated a controllable TiO_2_ coating on the LCO surface by a magnetron sputtering method [17]. The surface-modified LCO exhibited a high-rate capability and good cycling performance due to the critical role of TiO_2_ coating on the resistance of interfacial side reactions. However, the above surface-modified strategies for LCO contain additional high-temperature annealing processes, which inhibit the practical application of TiO_2_ coating. What is more, the thickness of coated TiO_2_ cannot be precisely controlled using the above methods. Therefore, it is necessary to develop a simple and controllable coating strategy of the TiO_2_ layer on LCO for a large-scale application.

In this work, an ultra-thin TiO_2_ nano-coating was applied to the surface-modification of LCO using atomic layer deposition (ALD) without additional annealing. The coating thickness of the TiO_2_ layer was precisely tuned by ALD technique. The ultra-thin TiO_2_ nano-coating enabled the modified LCO to exhibit a high-rate capability and significantly improved the cycling stability. Various characterizations, such as in situ XRD and ex situ XPS, were performed to reveal the origin of the improved electrochemical performance of surface-modified LCO. The ultra-thin TiO_2_ coating mitigates the side reactions at the electrode/electrolyte interface and forms a dense and stable CEI, which reduces the inhomogeneous Li^+^ transport and provides an effective channel for Li^+^ transport, thus improving the rate capability. Moreover, the protective layer can restrict crack formation, increasing the long-cycling stability. This oxide-coating modification provides a promising strategy to achieve high-stability HV-LCO.

## 2. Materials and Methods

Chemicals. All chemicals, including titanium tetrakis(dimethylamide) (TDMAT, 99.9999%, Nanjing Aimouyuan Scientific Equipment Co., Ltd., Nanjing, China), LiCoO_2_ (Guangdong Canrd New Energy Technology Co. Ltd., Dongguan, China), N-methyl pyrrolidone (NMP, 99.9%, Aladdin Biochemical Technology Co., Ltd., Shanghai, China), polyvinylidene fluoride (PVDF, Solvay (Shanghai) Co., Ltd., Shanghai, China) and Super P (Timcal Super C65, Imerys Graphite & Carbon, Shanghai, China), were used as received.

Sample Synthesis. The TiO_2_ layer was coated on LCO using the ALD technique, with TDMAT as the Ti source and H_2_O as the oxygen source. Typically, LCO powder was loaded in the ALD reaction chamber. After the reactor temperature reached 170 °C, the ALD cycle started with vapors of precursors pulsing in the chamber as follows: (1) TDMAT for 100 ms; (2) N_2_ for 20 s; (3) H_2_O for 20 ms; (4) N_2_ for 25 s. The coated samples were achieved after 25, 50 and 100 ALD cycles, denoted as LCO-ALD25, LCO-ALD50 and LCO-ALD100, respectively.

Characterization. The phase and crystal structure of the samples were characterized on an Ultima IV powder X-ray diffractometer (Rigaku Co., Ltd., Tokyo, Japan) with Cu Kα radiation (λ = 1.5406 Å) at a scanning speed of 10° min^−1^. Rietveld refinement of the X-ray diffraction (XRD) pattern was conducted using the General Structure Analysis System (GSAS) Program (Version 1.00). Morphologies and compositions of the samples were characterized by using field-emission scanning electron microcopy (FESEM, JSM-7800F, JEOL (Beijing) Co., Ltd., Beijing, China) equipped with an energy dispersive function at an accelerating voltage of 10 kV. The elemental distribution of the samples was characterized by using energy dispersive spectroscopy (EDS, 51-XMX1236, Oxford Instrument Technology (Shanghai) Co., LTD, Shanghai, China). High-resolution transmission electron microscope (HRTEM) images were collected on a JEM-2100Plus (JEOL (Beijing) Co., Ltd., Beijing, China) at an accelerating voltage of 200 kV. Surface analysis was performed on an X-ray photoelectron spectroscope (XPS, PHI5000 Ver-saprobe III, ULVAC-PHI, Inc. Kanagawa, Japan) with an X-ray source of Al.

Electrochemical measurement. The electrochemical performances of the LCO-ALD25, LCO-ALD50, LCO-ALD100 and bare LCO samples were tested using standard CR2032 coin cells. The working electrodes were prepared with the active material, Super P, and PVDF in a mass ratio of 80:10:10. These electrode ingredients were dispersed in NMP. The obtained slurry was painted on the aluminum foil and dried in a vacuum oven at 110 °C for 12 h. The mass loading of electrodes is about 3.1 mg cm^−2^. The cells were assembled in an argon-filled glovebox with O_2_ and H_2_O levels below 0.1 ppm. Lithium metal foil was used as the counter electrode. A total of 1 M LiPF_6_ dissolved in ethylene carbonate/dimethyl carbonate/ethylene methyl carbonate (EC/DMC/EMC, *v*/*v*/*v*, 1/1/1) with 5% fluoroethylene carbonate (FEC) was used as the electrolyte. Galvanostatic charge-discharge (GCD) was performed using a Neware instrument (CT-4008) over a voltage range of 3 to 4.5 V. Cyclic voltammetry (CV) curves were collected by the Bio-Logic electrochemical workstation (SP-150, Bio-Logic SAS) at different scan rates over a voltage range of 3 to 4.5 V. The electrochemical workstation was also used to measure for electrochemical impedance spectroscopy (EIS) with an AC stimulus of 10 mV and amplitude frequencies in the range of 100 kHz to 0.01 Hz.

## 3. Results and Discussion

**Figure 1:**
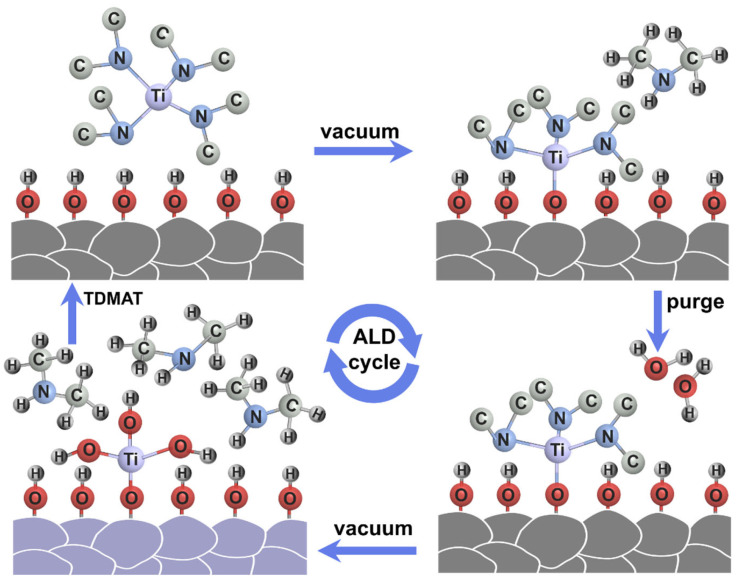
Schematic illustration of the preparation procedure of the TiO_2_ layer on LCO by ALD.

Figure 1 schematically illustrates the preparation procedure of the TiO_2_ layer by ALD method. After an initial water vapor bath pulse to hydroxylate the surface, TDMAT, which is a titanium precursor, is forced into the reaction chamber. TDMAT reacts directly with the hydroxyl groups of the LiCoO_2_ particles to form Ti-O-CH_3_ and NH-C_2_H_6_. Excess titanium precursor and NH-C_2_H_6_ are removed by vacuum. In parallel with the water vapor purge, as a source of oxygen reaction, the powder is placed in steam to hydrolyze LiCoO_2_ and the surface layer of the titanium precursor Ti-O-CH_3_ so as to form -Ti-OH and NH-C_2_H_6_. By repeating this sequence, TiO_2_ coating layers could be gradually formed on LiCoO_2_ particles.

**Figure 2:**
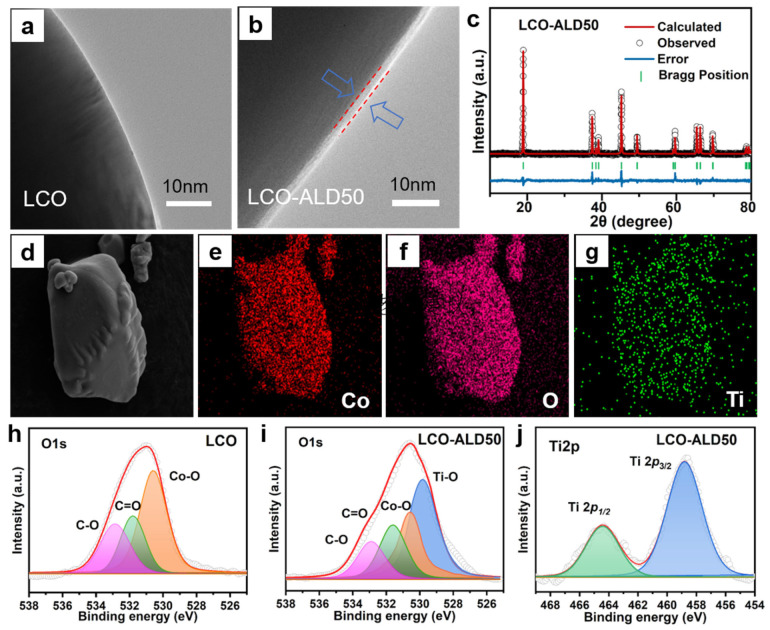
TEM images of (**a**) bare LCO and (**b**) LCO-ALD50 after TiO_2_ coating. (**c**) XRD patterns of LCO-ALD50 with Rietveld refinement. (**d**) FESEM image of LCO-ALD50 and the corresponding EDS images with elements (**e**) Co, (**f**) O and (**g**) Ti. High-resolution O1s XPS spectra of bare (**h**) LCO and (**i**) LCO-ALD50. (**j**) High-resolution Ti2p XPS spectra of LCO-ALD50.

The TEM images of bare LCO and LCO-ALD50 are shown in Figure 2a,b and Figure S1. The bare LCO exhibits a smooth morphology on the surface (Figure 2a). In contrast, the surface of LCO-ALD50 has a uniform coating layer with a thickness of about 2–3 nm (Figure 2b). As for the high-resolution TEM image (Figure S1), the bulk structure of LCO maintains a layered structure with an interplane spacing of ~0.468 nm, corresponding to the (003) plane of LCO. The coating layer on LCO-ALD50 exhibits an obvious amorphous structure that is ascribed to the coated TiO_2_ by ALD method. Figure S2 compares the Raman spectra of bare LCO and LCO-ALD50. Obviously, there are no new peaks appearing after surface-coating for LCO-ALD50, further demonstrating the amorphous TiO_2_ coating. XRD patterns of bare LCO, LCO-ALD25, LCO-ALD50 and LCO-ALD100 are shown in Figure S3. All the diffraction peaks of surface-modified LCO are in agreement with the standard card (Power Diffraction File No. 50-0653) without any impurities after coating TiO_2_ [20]. The peaks (018) and (110) of bare LCO and surface-modified LCO are well separated, demonstrating the highly ordered layered structure [21]. Based on the crystallographic data of LCO, the crystal structures of bare LCO, LCO-ALD25, LCO-ALD50 and LCO-ALD100 were analyzed using the XRD Rietveld refinement method (Figure 2c and Figures S4–S6). The crystallographic parameters and layer spacing are shown in Table S1. Lattice parameters “a” and “c” of surface-modified LCO (a = 2.8177 Å, c = 14.0648 Å) increased slightly compared to bare LCO (a = 2.8157 Å, c = 14.0379 Å), which can be ascribed to the O loss during the formation of Ti-O-CH_3_ in the ALD cycles. The increased c/a layer spacing can provide more sites to accommodate Li^+^ [22], benefiting the high capacities of LCO-ALD25, LCO-ALD50 and LCO-ALD100.

Figure 2d–g exhibit the FESEM image and corresponding energy-dispersive X-ray spectroscopy (EDS) images of LCO-ALD50. There is a uniform distribution of Co, O, and Ti elements, indicating the successful coating of the TiO_2_ layer on the LCO-ALD50 surface. The O1s high-resolution XPS spectra of bare LCO and LCO-ALD50 are shown in Figure 2h,i, respectively. The deconvoluted peaks at 532.8, 531.8 and 530.6 eV can be ascribed to the C-O, C=O and Co-O, respectively [4,15]. There is a new deconvoluted peak at 529.8 eV for LCO-ALD50, corresponding to Ti-O from the coated TiO_2_ layer [23]. Figure 2j shows the high-resolution Ti2p XPS spectrum of LCO-ALD50. There are orbital spin-splitting peaks at 458.9 eV and 464.6 eV, which are ascribed to Ti2p_1/2_ and Ti2p_3/2_, respectively [24], further confirming the presence of the TiO_2_ layer.

**Figure 3:**
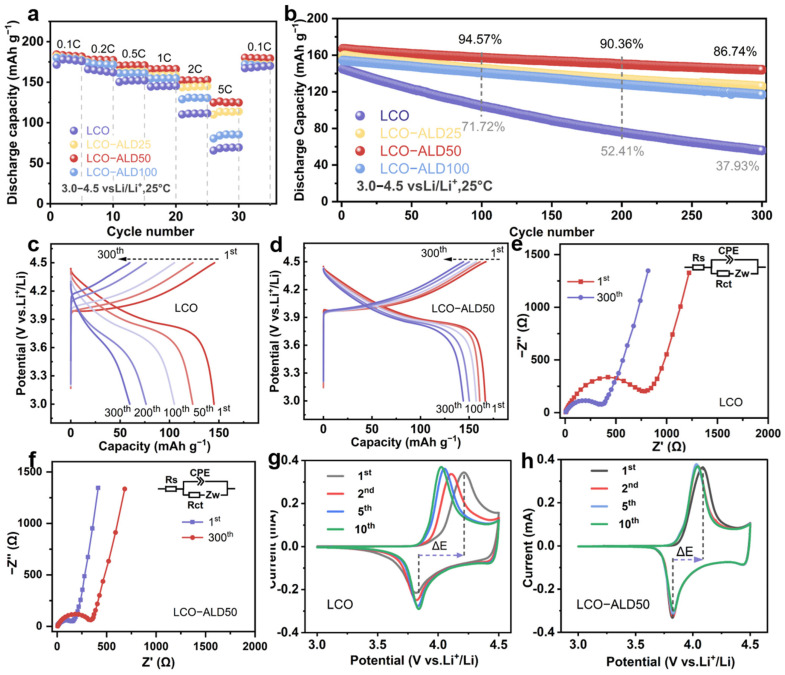
Electrochemical properties. (**a**) Rate capability at different C-rates and (**b**) cycling stability at 1C (1C = 274 mAh g^−1^) of bare LCO, LCO-ALD25, LCO-ALD50, and LCO-ALD100. The 1st, 50th, 100th, 200th and 300th galvanostatic charge/discharge profiles of (**c**) bare LCO and (**d**) LCO-ALD50 at 1C. (**e**) The comparison of cycling performance between LCO-ALD50 and the reported TiO_2_-coated LCO electrodes. (**f**) Nyquist plots before and after 300 cycles of LCO-ALD50. The 1st, 2nd, 5th and 10th CV curves of (**g**) bare LCO and (**h**) LCO-ALD50 measured at 0.1 mV s^−1^.

To analyze the effect of the TiO_2_ coating strategy on the electrochemical performance of LCO, several electrochemical tests were performed on the half-cells in the voltage range of 3.0–4.5 V using bare LCO and surface-modified LCO as working electrodes. Figure 3a compares the rate performance of all samples. The LCO-ALD50 electrode exhibits the best rate capability among all samples, maintaining reversible discharge capacities of 180, 177, 171, 166, and 152 mAh g^−1^ at 0.1, 0.2, 0.5, 1, and 2C (1C = 274 mAh g^−1^), respectively. Even at a high rate of 5C, it also delivered a very impressive capacity of 125 mAh g^−1^, much larger than that of bare LCO (66 mAh g^−1^). The enhanced rate performance of LCO-ALD50 is ascribed to the uniform and suitable surface coating of TiO_2_. When reverted from 5C to 0.1C, the specific capacity remained almost constant for LCO-ALD50, indicating the high reversibility against cycling.

Figure 3b compares the long cycling performance of bare LCO and surface-modified LCO electrodes at 1C. Notably, LCO-ALD50 exhibits excellent stability against cycling with an 86.7% capacity retention after 300 cycles, beyond that of bare LCO (37.9%). Figure 3c,d show the selected charge/discharge profiles of bare LCO and LCO-ALD50 at 1C. Obviously, LCO-ALD50 maintains stable voltage profiles with little polarization. In contrast, bare LCO displays a rapid capacity fading due to structural degradation. The excellent cycling stability is ascribed to the ultra-thin TiO_2_ protective layer and stable CEI, which can decrease the polarization during the charge/discharge process and suppress side reactions, respectively. Figure 3e shows a comparison of the cycling performance for LCO-ALD50 with the reported TiO_2_-coated LCO electrodes at 4.3–4.5 V [17,18,19,25,26,27,28]. Obviously, the LCO-ALD50 electrode in this work exhibits a better long cycling stability than that of others, further demonstrating the positive effect of amorphous TiO_2_ in regulating electrode–electrolyte interface compatibility.

The Nyquist plots of bare LCO and LCO-ALD50 with the equivalent circuit models are shown in Figure 3e and Figure S7. The fitting results are shown in Table S2. The intercept of the curve with the x-axis is the internal resistance (*R*_s_) of the electrolyte and the electrode [29,30]. The semicircle and the diagonal line indicate the charge transfer resistance (*R*_ct_) and the Warburg impedance (*Z*_w_) [31]. The *R*_ct_ values of LCO-ALD50 before cycling and after 300 cycles are determined to be 352.4 and 189.2 Ω, respectively, which are smaller than those of bare LCO (863.2 and 382.7 Ω). The smaller *R*_ct_ indicates less resistance to mass transfer at the interface of electrode/electrolyte, which can be attributed to the reduced surface structural degradation because of TiO_2_ coating [32]. Figure 3g,h show the cyclic voltammetry (CV) curves for the first ten cycles of bare LCO and LCO-ALD50 measured at 0.1 mV s^−1^. There is a pair of redox peaks at about 3.8 and 4.1 V for both samples, corresponding to the redox reactions of Co^3+^/Co^4+^ during Li^+^ insertion and extraction [5]. The LCO-ALD50 electrode exhibits a lower polarization voltage (ΔE = 0.20 V) in the first cycle than that of the bare LCO electrode (ΔE = 0.37 V). What is more, the CV curves almost overlap throughout the cycles, further confirming the good electrochemical reversibility of the LCO-ALD50 electrode.

The diffusion of Li^+^ in bare LCO and LCO-ALD50 was investigated using the constant current intermittent titration technique (GITT), and the results are shown in Figures S8 and S9. The GITT curves for different states were calculated according to Fick’s second law. The average Li^+^ diffusion coefficients of LCO-ALD50 reached 2.82 × 10^−11^ (charging) and 2.18 × 10^−11^ cm^2^ s^−1^ (discharging), higher than those of LCO (1.54 × 10^−11^ cm^2^ s^−1^ for charging and 1.12 × 10^−11^ cm^2^ s^−1^ for discharging) (Table S3). These results indicate that LCO-ALD50 has a rapid Li^+^ (de)intercalation after surface modification.

**Figure 4:**
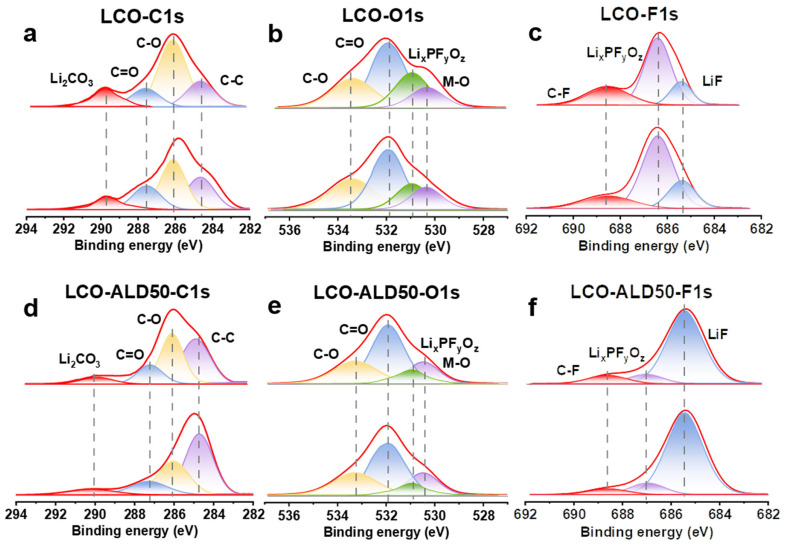
Investigation of the excellent surface stability of bare LCO and LCO-ALD50 at 4.5 V. XPS depth spectra of (**a**) C1s, (**b**) O1s, and (**c**) F1s for bare LCO and of (**d**) C1s, (**e**) O1s, and (**f**) F1s for LCO-ALD50 after 100 cycles at 1C.

Figure 4a–f exhibit the XPS depth spectra of bare LCO and LCO-ALD50 after 100 cycles at 4.5 V to investigate the dynamic evolution of CEI. The deconvoluted peaks at 286.3 eV and 287.6 eV in the C1s spectra (Figure 4a,d) correspond to C-O and C=O, respectively [4]. Additionally, the peaks at 533.4 eV and 532.0 eV in the O1s spectra (Figure 4b,e) are ascribed to C-O and C=O, respectively [6,14]. Comparing the C-O and C=O peaks in the C1s and O1s spectra, the bare LCO exhibits a higher intensity than the LCO-ALD50 due to the decomposition of the electrolyte [32]. In addition, the content of Li_2_CO_3_ (589.8 eV) in the C1s spectra of bare LCO is higher than that of LCO-ALD50, suggesting that the bare LCO has a greater reduction by the carbonate solvent during charging and discharging [20]. The deconvoluted peaks at 530.9 eV and 530.3 eV in the O1s spectra can be ascribed to Li_x_PF_y_O_z_ and M-O, respectively [7,12]. The intensity of M-O can be used as an indicator of the thickness of the CEI layer. When the etching time is 0 s, the intensity of the M-O peaks for bare LCO is lower than that of LCO-ALD50, indicating that bare LCO has a faster CEI growth during the cycling process. For LCO-ALD50, the M-O peaks are almost constant with the etching time, indicating the formation of a uniform and dense CEI [33]. In both O1s (Figure 4b,e) and F1s (Figure 4c,f), there are Li_x_PF_y_O_z_ peaks (686.4 eV), which are ascribed to LiPF_6_ decomposition [4]. The intensity of Li_x_PF_y_O_z_ peaks of LCO-ALD50 is lower than that of bare LCO, suggesting that the passivated CEI hinders the decomposition of LiPF_6_ during charge/discharge cycling. The deconvolution peaks at 685.3 eV in the F1s spectra (Figure 4c,f) can be ascribed to LiF [4,13]. ALD50-LCO exhibits a higher LiF content than bare LCO. The high density of CEI and high content of LiF are required to avoid the decomposition of the electrolyte and rapid dissolution of Li^+^ during the intercalation process [34]. Therefore, ALD50-LCO exhibits a high-rate performance and excellent long-term stability.

The structural evolution of bare LCO and ALD50-LCO during charging and discharging was investigated using in situ XRD, and the results are shown in Figures S10 and S11. In general, the evolution of the (003) diffraction peaks can reflect the changes in the layer structure [35], and the shift of the peak position is related to the volume expansion [36]. It can be seen that the (003) peaks of both bare LCO and LCO-ALD50 were shifted at a lower angle during the charging process, indicating that a phase transition from H1 to H3 occurred during Li^+^ insertion. Compared to bare LCO, LCO-ALD50 exhibits a lower peak shift and has a more continuous structural change when charged to 4.5 V, indicating a smaller volume change in favor of structural anti-cycling stability.

**Figure 5:**
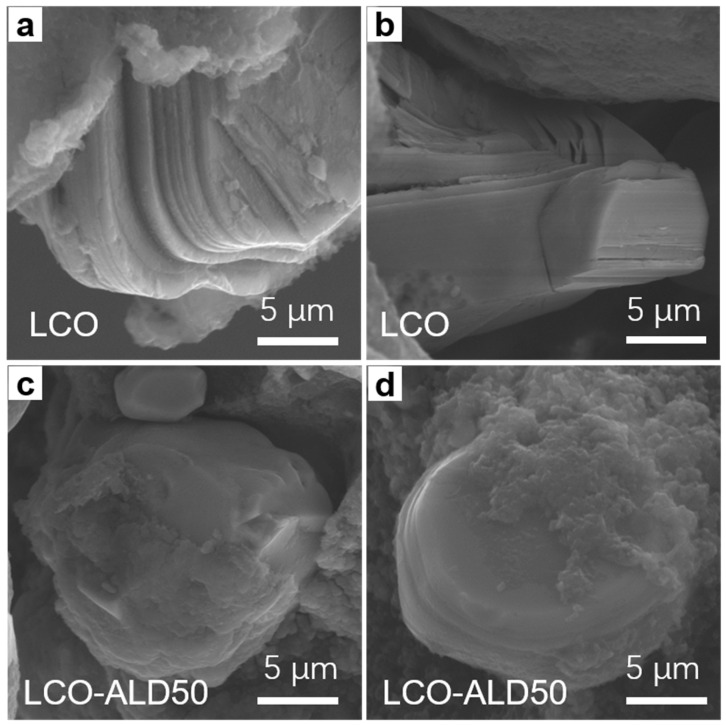
FESEM images of (**a**,**b**) bare LCO and (**c**,**d**) LCO-ALD50 after 300 cycles at 1C.

To reveal the structural stability after charging cycles, the cross-sectional morphologies of bare LCO and LCO-ALD50 after 300 cycles at 1C were obtained by SEM measurements. As shown in Figure 5a,b, bare LCO suffers from severe cracks from the surface to the bulk and obvious interlayer slipping. In contrast, no appreciable cracks can be found in LCO-ALD50 (Figure 5c,d). For LCO-ALD50 cathode materials, the phase change is reversible and the internal stress can be reduced.

According to the above results on physical characterization and electrochemical performance, it can be found that the CEI of bare LCO involves a dynamic evolution of regeneration due to the electrolyte’s decomposition, resulting in a sluggish Li^+^ transformation. In contrast, a dense and stable CEI forms on LCO-ALD50 through the surface stabilization of TiO_2_, benefitting a fast Li^+^ transportation and enhancing stability against long cycling. Thus, LCO-ALD50 exhibits an excellent high-rate capability and long-life cyclability.

## 4. Conclusions

In summary, an ultra-thin and uniform nano-layer of TiO_2_ was precisely deposited on the surface of LCO by ALD technique. The surface-modified LCO exhibits an enhanced electrochemical performance. The optimized TiO_2_-coated LCO (LCO-ALD50) exhibits a high-rate capacity of 125 mAh g^−1^ at 5C and an improved cycling stability with a high-capacity retention (86.7%) after 300 cycles at 1C, far outperforming the bare LCO (37.9%). The in situ XRD and ex situ XPS results reveal that the ultra-thin TiO_2_ coating can mitigate the side reactions at the electrode/electrolyte interface and forms a dense and stable CEI, which reduces the inhomogeneous Li^+^ transport and provides an effective channel for Li^+^ transport, thus improving the rate capability. Moreover, the protective layer can restrict crack formation, increasing the long-cycling stability. This work provides a simple, efficient way to improve the electrochemical performance of HV-LCO for high-energy-density LIBs.

## Data Availability

Data are contained within the article.

## Supplementary Materials

The following supporting information can be downloaded at: https://www.mdpi.com/article/10.3390/ma17123036/s1, Figure S1: XRD patterns of bare LCO, LCO-ALD25, LCO-ALD50 and LCO-ALD100; Figure S2: XRD pattern with Rietveld refinement of bare LCO; Figure S3: XRD patterns with Rietveld refinement of LCO-ALD25; Figure S4: XRD patterns with Rietveld refinement of LCO-ALD100; Figure S5: GITT curve and variations in apparent Li^+^ diffusion coefficient of LCO; Figure S6: GITT curve and variations in apparent Li^+^ diffusion coefficient of LCO-ALD50; Figure S7: In situ XRD patterns during the first cycle of LCO; Figure S8: In situ XRD patterns during the first cycle of LCO-ALD50; Table S1: Structural information of bare LCO and surface-modified LCO obtained from the XRD Rietveld refinement; Table S2: Impedance parameters obtained from equivalent circuit fittings; Table S3: Diffusion coefficients of Li^+^.

## References

[1] Lin C., Li J., Yin Z.W., Huang W., Zhao Q., Weng Q., Liu Q., Sun J., Chen G., Pan F. (2024). Structural Understanding for High-Voltage Stabilization of Lithium Cobalt Oxide. Adv. Mater..

[2] Zhang H., Liu H., Piper L.F.J., Whittingham M.S., Zhou G. (2022). Oxygen Loss in Layered Oxide Cathodes for Li-Ion Batteries: Mechanisms, Effects, and Mitigation. Chem. Rev..

[3] Konar R., Maiti S., Shpigel N., Aurbach D. (2023). Reviewing Failure Mechanisms and Modification Strategies in Stabilizing High-Voltage LiCoO_2_ Cathodes beyond 4.55V. Energy Storage Mater..

[4] Zhuang Z., Wang J., Jia K., Ji G., Ma J., Han Z., Piao Z., Gao R., Ji H., Zhong X. (2023). Ultrahigh-Voltage LiCoO_2_ at 4.7 V by Interface Stabilization and Band Structure Modification. Adv. Mater..

[5] Wu N., Zhang Y., Guo Y., Liu S., Liu H., Wu H. (2016). Flakelike LiCoO_2_ with Exposed {010} Facets As a Stable Cathode Material for Highly Reversible Lithium Storage. ACS Appl. Mater. Interfaces.

[6] Xu S., Tan X., Ding W., Ren W., Zhao Q., Huang W., Liu J., Qi R., Zhang Y., Yang J. (2023). Promoting Surface Electric Conductivity for High-Rate LiCoO_2_. Angew. Chem. Int. Ed..

[7] Zhang J., Wong D., Zhang Q., Zhang N., Schulz C., Bartkowiak M., An K., Gu L., Hu Z., Liu X. (2023). Reducing Co/O Band Overlap through Spin State Modulation for Stabilized High Capability of 4.6 V LiCoO_2_. J. Am. Chem. Soc..

[8] Takamatsu D., Orikasa Y., Mori S., Nakatsutsumi T., Yamamoto K., Koyama Y., Minato T., Hirano T., Tanida H., Arai H. (2015). Effect of an Electrolyte Additive of Vinylene Carbonate on the Electronic Structure at the Surface of a Lithium Cobalt Oxide Electrode under Battery Operating Conditions. J. Phys. Chem. C.

[9] Zhang J.N., Li Q., Ouyang C., Yu X., Ge M., Huang X., Hu E., Ma C., Li S., Xiao R. (2019). Trace Doping of Multiple Elements Enables Stable Battery Cycling of LiCoO_2_ at 4.6 V. Nat. Energy.

[10] Wang Y., Zhang Q., Xue Z.C., Yang L., Wang J., Meng F., Li Q., Pan H., Zhang J.N., Jiang Z. (2020). An In Situ Formed Surface Coating Layer Enabling LiCoO_2_ with Stable 4.6 V High-Voltage Cycle Performances. Adv. Energy Mater..

[11] Zhang C., Shen X., Li X., Liu Q., Liu Z., Huang Y., Gao Y., Hu Z., Chen J.M., Yang Y. (2023). Quenching-Etched Surface Spinel to Passivate Layered Cathode Materials from Structural Degradation at High Potentials. Chem. Mater..

[12] Zhou H., Izumi J., Asano S., Ito K., Watanabe K., Suzuki K., Nemoto F., Yamada N.L., Aso K., Oshima Y. (2023). Fast Lithium Intercalation Mechanism on Surface-Modified Cathodes for Lithium-Ion Batteries. Adv. Energy Mater..

[13] Wu R., Cao T., Liu H., Cheng X., Liu X., Zhang Y. (2022). Ultralong Lifespan for High-Voltage LiCoO_2_ Enabled by in Situ Reconstruction of an Atomic Layer Deposition Coating Layer. ACS Appl. Mater. Interfaces.

[14] Dong W., Ye B., Cai M., Bai Y., Xie M., Sun X., Lv Z., Huang F. (2023). Superwettable High-Voltage LiCoO_2_ for Low-Temperature Lithium Ion Batteries. ACS Energy Lett..

[15] Wang Z., Dai X., Chen H., Wu F., Mai Y., Li S., Gu Y., Li J., Zhou A. (2022). Simultaneously Constructing a TiO_2_-LiF Composite Coating Enhancing the Cycling Stability of LiCoO_2_ at 4.6 V High Voltage. ACS Sustain. Chem. Eng..

[16] Xu L., Cheng S., Niu H., Wang Z. (2024). Understanding the Role of TiO_2_ Coating for Stabilizing 4.6V High-Voltage LiCoO_2_ Cathode Materials. Electrochim. Acta.

[17] Zhou A., Lu Y., Wang Q., Xu J., Wang W., Dai X., Li J. (2017). Sputtering TiO_2_ on LiCoO_2_ Composite Electrodes as a Simple and Effective Coating to Enhance High-Voltage Cathode Performance. J. Power Sources.

[18] Cho Y., Eom J., Cho J. (2010). High Performance LiCoO_2_ Cathode Materials at 60 °C for Lithium Secondary Batteries Prepared by the Facile Nanoscale Dry-Coating Method. J. Electrochem. Soc..

[19] Moon S.H., Kim M.C., Kim E.S., Shin Y.K., Lee J.E., Choi S., Park K.W. (2019). TiO_2_-Coated LiCoO_2_ Electrodes Fabricated by a Sputtering Deposition Method for Lithium-Ion Batteries with Enhanced Electrochemical Performance. RSC Adv..

[20] Ren H., Zhao W., Yi H., Chen Z., Ji H., Jun Q., Ding W., Li Z., Shang M., Fang J. (2023). One-Step Sintering Synthesis Achieving Multiple Structure Modulations for High-Voltage LiCoO_2_. Adv. Funct. Mater..

[21] Huang Y., Zhu Y., Fu H., Ou M., Hu C., Yu S., Hu Z., Chen C.T., Jiang G., Gu H. (2021). Mg-Pillared LiCoO_2_: Towards Stable Cycling at 4.6 V. Angew. Chem. Int. Ed..

[22] Chen J., Chen H., Zhang S., Dai A., Li T., Mei Y., Ni L., Gao X., Deng W., Yu L. (2022). Structure/Interface Coupling Effect for High-Voltage LiCoO_2_ Cathodes. Adv. Mater..

[23] Wang C., Zhang J., Wang X., Lin C., Zhao X.S. (2020). Hollow Rutile Cuboid Arrays Grown on Carbon Fiber Cloth as a Flexible Electrode for Sodium-Ion Batteries. Adv. Funct. Mater..

[24] Jia R., Zhang R., Yu L., Kong X., Bao S., Tu M., Liu X., Xu B. (2023). Engineering a Hierarchical Carbon Supported Magnetite Nanoparticles Composite from Metal Organic Framework and Graphene Oxide for Lithium-Ion Storage. J. Colloid Interface Sci..

[25] Ting-Kuo Fey G., Lu C.Z., Prem Kumar T., Chang Y.C. (2005). TiO_2_ Coating for Long-Cycling LiCoO_2_: A Comparison of Coating Procedures. Surf. Coat. Technol..

[26] Su L., Jha S.K., Phuah X.L., Xu J., Nakamura N., Wang H., Okasinski J.S., Reeja-Jayan B. (2020). Engineering Lithium-Ion Battery Cathodes for High-Voltage Applications Using Electromagnetic Excitation. J. Mater. Sci..

[27] Cheng H.M., Wang F.M., Chu J.P., Santhanam R., Rick J., Lo S.C. (2012). Enhanced Cycleabity in Lithium Ion Batteries: Resulting from Atomic Layer Depostion of Al_2_O_3_ or TiO_2_ on LiCoO_2_ Electrodes. J. Phys. Chem. C.

[28] Jayasree S.S., Nair S., Santhanagopalan D. (2018). Ultrathin TiO_2_ Coating on LiCoO_2_ for Improved Electrochemical Performance as Li–Ion Battery Cathode. ChemistrySelect.

[29] Wang Q., Wang C., Zheng K., Wang B., Wang Z., Zhang C., Long X. (2024). Positional Thiophene Isomerization: A Geometric Strategy for Precisely Regulating the Electronic State of Covalent Organic Frameworks to Boost Oxygen Reduction. Angew. Chem. Int. Ed..

[30] Shin B.H., Kim S., Park J., Ok J.W., Kim D.I., Kim D., Yoon J.H. (2024). Effect of Secondary Phase on Electroless Ni Plating Behaviour of Super Duplex Stainless Steel SAF2507 for Advanced Li-Ion Battery Case. Materials.

[31] Xiao Z., Gao L., Li S. (2024). Engineering Heterostructured Fe-Co-P Arrays for Robust Sodium Storage. Materials.

[32] Yang X., Wang C., Yan P., Jiao T., Hao J., Jiang Y., Ren F., Zhang W., Zheng J., Cheng Y. (2022). Pushing Lithium Cobalt Oxides to 4.7 V by Lattice-Matched Interfacial Engineering. Adv. Energy Mater..

[33] Li J., Lin C., Weng M., Qiu Y., Chen P., Yang K., Huang W., Hong Y., Li J., Zhang M. (2021). Structural Origin of the High-Voltage Instability of Lithium Cobalt Oxide. Nat. Nanotechnol..

[34] Zheng J., Wang Y., Qin M., Sun L., Peng C., Li Y., Feng W. (2023). Epitaxial Growth of a Single Hexagonal Layered α-LiAlO_2_ Coating on a High-Voltage LiCoO_2_ Cathode Material for Enhanced Stability. J. Mater. Chem. A.

[35] Wang P., Meng Y., Wang Y., Chen L., Zhang Z., Pu W., Li J., Yang C., Xiao D. (2022). Oxygen Framework Reconstruction by LiAlH_4_ Treatment Enabling Stable Cycling of High-Voltage LiCoO_2_. Energy Storage Mater..

[36] Li Y., Zan M., Chen P., Huang Y., Xu X., Zhang C., Cai Z., Yu X., Li H. (2023). Facile Solid-State Synthesis to In Situ Generate a Composite Coating Layer Composed of Spinel-Structural Compounds and Li_3_PO_4_ for Stable Cycling of LiCoO_2_ at 4.6 V. ACS Appl. Mater. Interfaces.

