# Supplementary material for: Ultrathin Titanium Dioxide Coating Enables High-Rate and Long-Life Lithium Cobalt Oxide
Source: Materials (Basel). 2024 Jun 20;17(12):3036. doi: 10.3390/ma17123036 (PMC11205603; doi:10.3390/ma17123036)
Supplement: Supplementary file 1 [file materials-17-03036-s001.zip › materials-3034476-supplementary.pdf]

Supporting Information

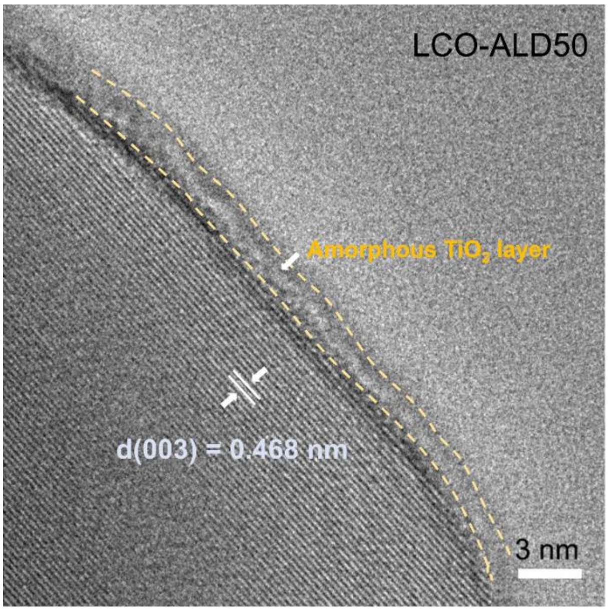

Figure S1. High-resolution TEM image of LCO-ALD50.

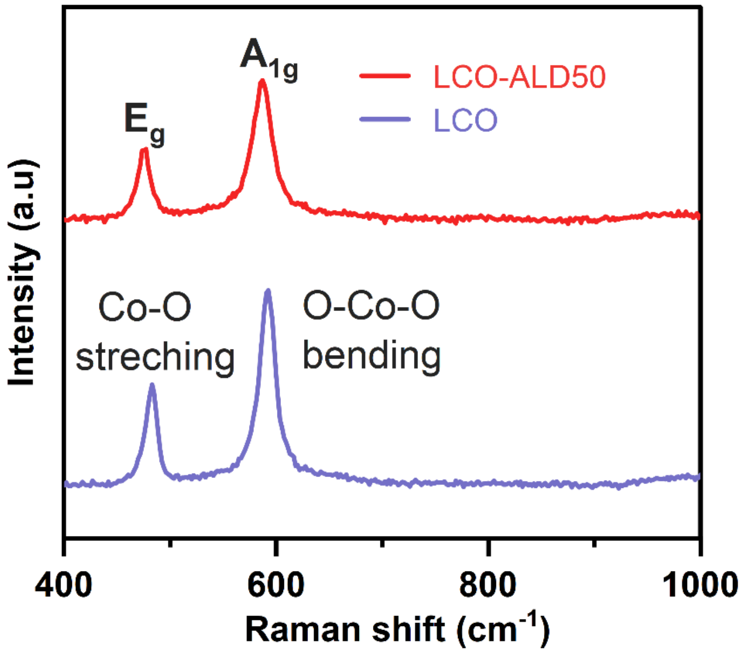

Figure S2. Raman spectra of bare LCO and LCO-ALD50.

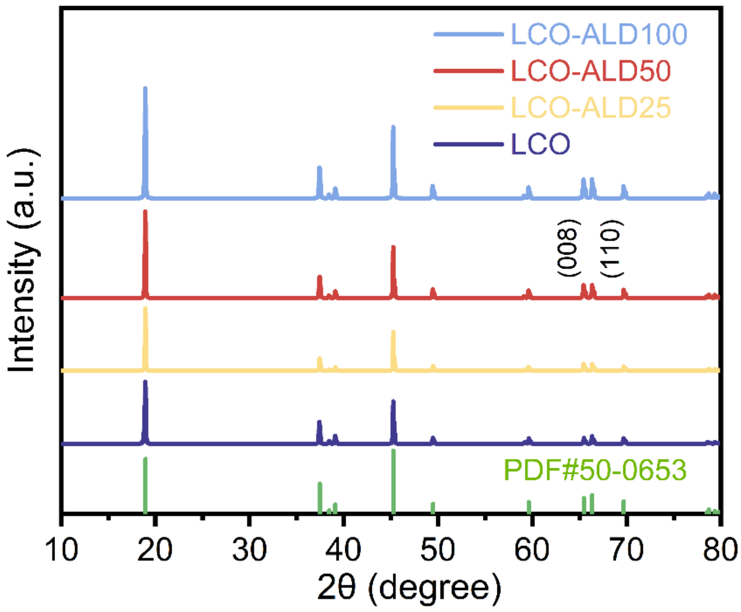

Figure S3. XRD patterns of bare LCO, LCO-ALD25, LCO-ALD50 and LCO-ALD100.

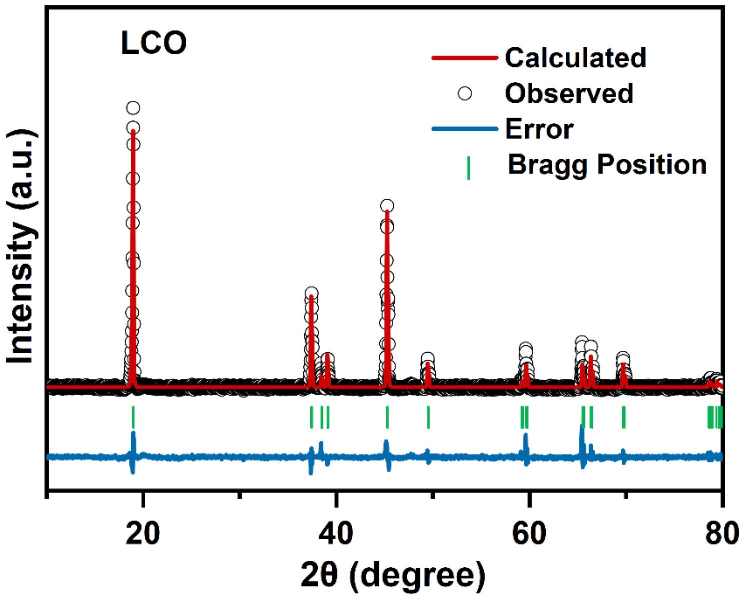

Figure S4. XRD pattern with Rietveld refinement of bare LCO.

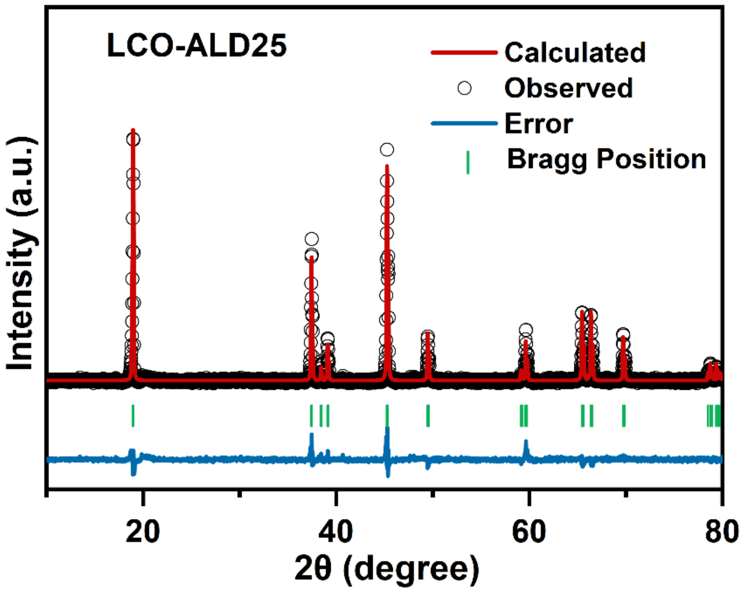

Figure S5. XRD patterns with Rietveld refinement of LCO-ALD25.

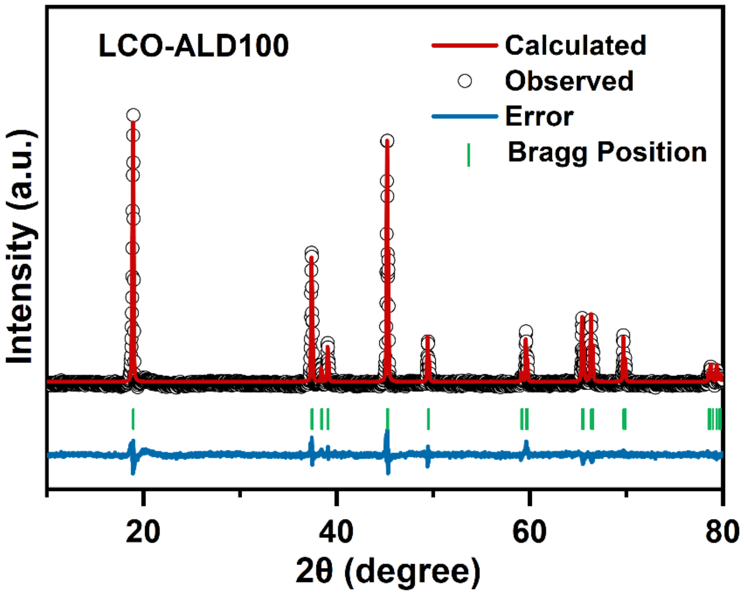

Figure S6. XRD patterns with Rietveld refinement of LCO-ALD100.

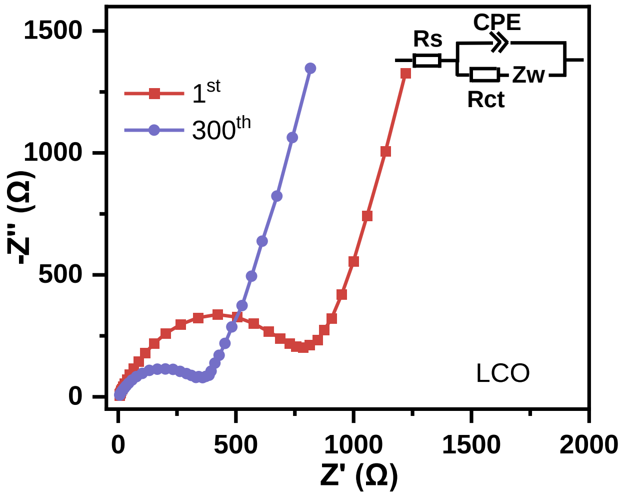

Figure S7. Nyquist plots before and after 300 cycles of bare LCO.

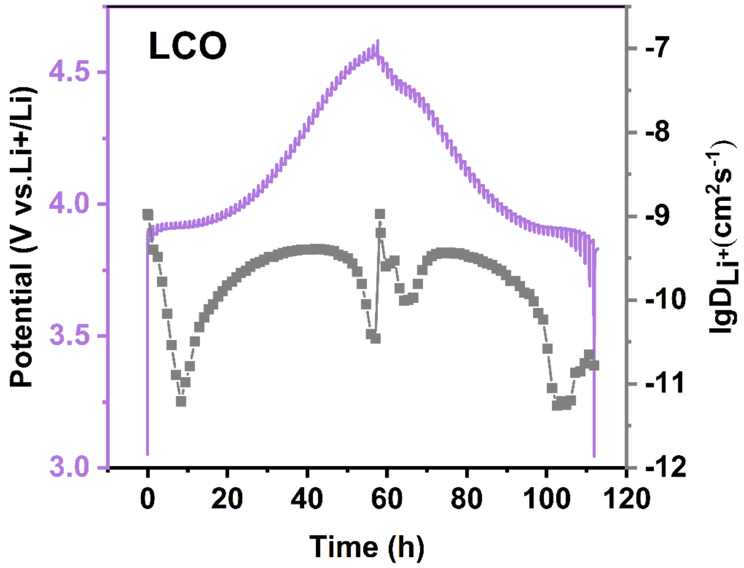

Figure S8. GITT curve and variations in apparent Li^+ diffusion coefficient of LCO.

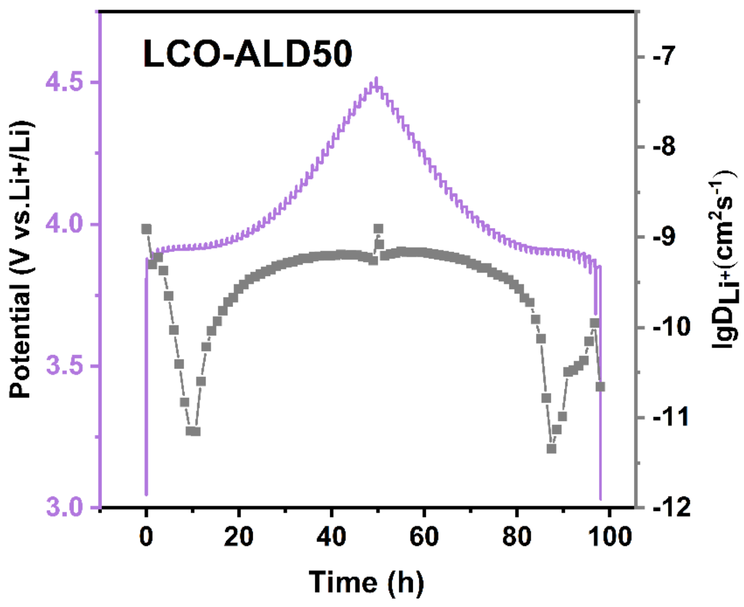

Figure S9. GITT curve and variations in apparent Li^+ diffusion coefficient of LCO-ALD50.

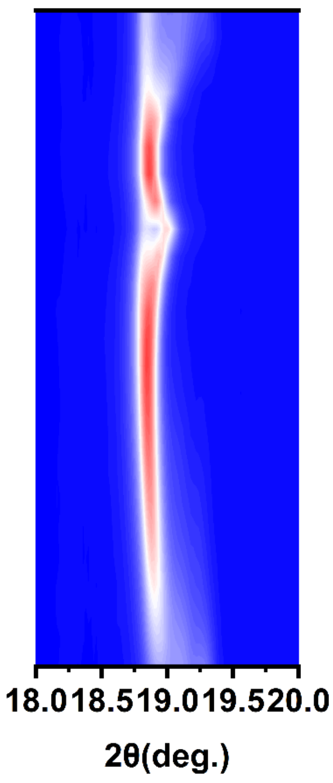

Figure S10. In situ XRD patterns during the first cycle of LCO.

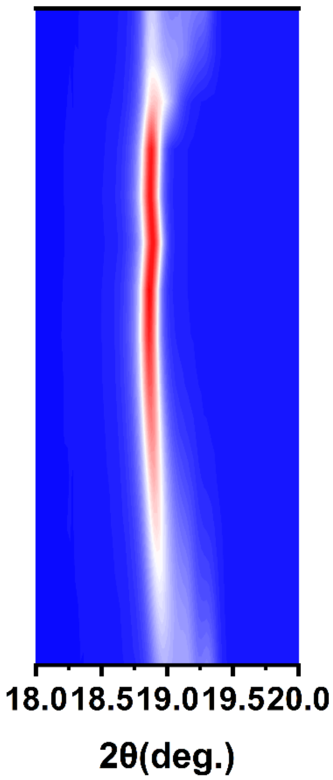

Figure S11. In situ XRD patterns during the first cycle of LCO-ALD50.

Table S1. Structural information of bare LCO and surface-modified LCO obtained from the XRD Rietveld refinement.

Sample	interlayer spacing		a	c	c/a
	003	104			
LCO	4.6793	2.0025	2.8157	14.0379	4.9855
ALD25	4.6881	2.0046	2.8175	14.0645	4.9914
ALD50	4.6883	2.0048	2.8177	14.0648	4.9917
ALD100	4.6882	2.0047	2.8176	14.0646	4.9916

Table S2. Impedance parameters obtained from equivalent circuit fittings.

Sample	R_{ct} (Ω)	Error (%)
LCO (before cycling)	863.2	6.4
LCO-ALD50 (before cycling)	352.4	7.9
LCO (after 100 cycles at 1C)	382.7	5.6
LCO-ALD50 (after 100 cycles at 1C)	189.2	6.1

Table S3. Diffusion coefficients of Li^+.

Samples	$D_{Li+}in$ / 10^{-11} cm^2s^{-1}	$D_{Li+}ex$ / 10^{-11} cm^2s^{-1}
Bare LCO	1.54	1.12
LCO-ALD50	2.82	2.18

$D_{Li+}in$: diffusion coefficient of Li+ during the charge process.

$D_{Li+}ex$: diffusion coefficient of Li+ during the discharge process.
